# Discipline in Convalescent Homes

**Published:** 1914-11-28

**Authors:** 


### Discipline in Convalescent Homes.

*To the Editor of* The Hospital.

Sir,—Your paragraph in the current issue of The Hospital headed "Discipline in Convalescent Homes" is of unusual interest to all engaged in convalescent home work, and especially to homes now nursing the wounded; but whether the heads of such institutions will agree with your remarks is open to doubt. You say, *inter alia*, "Patients should not be allowed greater freedom than in ordinary hospitals, and, as far as possible, when out for motor-drives and similar excursions they should be attended by orderlies from the male Voluntary Aid Detachments. Cases have occurred in which soldiers have been allowed to enter public-houses, or have been treated when out of hospital, and precautions must be taken to prevent any such infringement of the ordinary hospital regulations. This is the natural and inevitable view . . ." I must urge that this view is, in my opinion, neither natural nor inevitable. The function of the convalescent homes as Voluntary Aid Detachments is, at the present time, twofold: (1) to relieve the hospitals of all cases that can be safely taken by them, and (2) if possible to fit the men for active service, or failing this, to restore them to the highest state of efficiency possible. Now in nearly all hospitals the patients are either confined to the wards and their balconies or they may be allowed to go into the garden. Convalescent treatment properly applied allows the maximum amount of liberty possible to the patients, and to suggest confining them to the grounds of the institution is to invite insubordination. You are to imagine (if one follows the recommendations of the Red Cross Commissioner to their natural conclusion) the patients being built up with nourishing food and tonics, and when they wax fat and kick they are to have no freedom to allow them properly to apply their newly-found energy. How are these poor fellows to rejoin their depôts in anything like fighting condition if they are not allowed all the outdoor exercise they can take? Such restrictions, if applied, can only serve to undermine the discipline they are presumably designed to support. Again, how is it possible to send an orderly with every motor-car taking men for a drive? In the institution I serve we send off perhaps a dozen cars in the course of the day.

The question of the soldiers entering public-houses is a serious one; but even here the difficulty is not insurmountable. We have had under the roof at one time some 120 Belgian wounded, twenty-four English "Tommies," and a number of male civilians; and all the ordinary difficulties of discipline were multiplied tenfold by the foolish spoiling of the men by people in the town. All our patients who were able to get out were free to do so morning and afternoon up to 5 p.m. Those who broke the rules seriously were dealt with at once, and already a good understanding has arisen between the patients and ourselves—they know that so long as they do not try to take gross liberties they may enjoy themselves to the full. One Belgian at the very beginning was missing at tea-time, and was eventually brought in very late by the police. He was placed under arrest, and eventually returned under escort to the hospital which sent him. The well-behaved patients—the large majority—were indignant at his abuse of hospitality and the liberty allowed, and the others saw that we meant business. Public opinion properly directed is the most powerful aid to discipline, and this was made use of both in the above case and in that of an English soldier who had to be sent back to the officer commanding at the hospital he came from.

There is one other point to which I should like to draw your attention, though it has no bearing on your paragraph. An appeal is being made for a home for men who are suffering from nervous shock. Why not select one or two convalescent homes and utilise them for this purpose, thus making available what we have? The homes are for the most part situated in healthy, pleasant surroundings, and, speaking generally, the restful change and wholesome feeding combined with the necessary medical attention which these places afford would, I am convinced, quickly restore to the normal any ordinary nerve case. If other homes have done as we have the medical and surgical staffs have been augmented, and they are prepared to do much more serious work than is required of them in times of peace.—Yours truly,

A Convalescent Home Secretary.

November 24, 1914.

[Neither the Red Cross Commissioner referred to nor ourselves suggested that convalescent patients should be confined to hospital grounds, as the specific allusion to motor drives is enough to show. But the interesting experiences at his own institution which our correspondent gives are examples of the difficulty of combining liberty and discipline.—Ed. The Hospital.]

